# Multidisciplinary Pharmacotherapeutic Options for Nonalcoholic Fatty Liver Disease

**DOI:** 10.1155/2012/950693

**Published:** 2012-12-09

**Authors:** Kei Nakajima

**Affiliations:** Division of Clinical Nutrition, Department of Medical Dietetics, Faculty of Pharmaceutical Sciences, Josai University, 1-1 Keyakidai, Saitama, Sakado 350-0295, Japan

## Abstract

Nonalcoholic fatty liver disease (NAFLD) and non-alcoholic steatohepatitis (NASH) are multidisciplinary liver diseases that often accompany type 2 diabetes or metabolic syndrome, which are characterized by insulin resistance. Therefore, effective treatment of type 2 diabetes and metabolic syndrome should target not only the cardiometabolic abnormalities, but also the associated liver disorders. In the last decade, it has been shown that metformin, thiazolidinediones, vitamin E, ezetimibe, n-3 polyunsaturated fatty acids, renin-angiotensin system (RAS) blockers, and antiobesity drugs may improve hepatic pathophysiological disorders as well as clinical parameters. Accordingly, insulin sensitizers, antioxidative agents, Niemann-Pick C1-like 1 (NPC1L1) inhibitors, RAS blockers, and drugs that target the central nervous system may represent candidate pharmacotherapies for NAFLD and possibly NASH. However, the efficacy, safety, and tolerability of long-term treatment (potentially for many years) with these drugs have not been fully established. Furthermore, clinical trials have not comprehensively examined the efficacy of lipid-lowering drugs (i.e., statins, fibrates, and NPC1L1 inhibitors) for the treatment of NAFLD. Although clinical evidence for RAS blockers and incretin-based agents (GLP-1 analogs and dipeptidyl peptidase-4 inhibitors) is also lacking, these agents are promising in terms of their insulin-sensitizing and anti-inflammatory effects without causing weight gain.

## 1. Introduction

Over the past two decades, the prevalence of metabolic abnormalities such as type 2 diabetes and metabolic syndrome (MetS) has been increasing worldwide together with the escalating obesity pandemic [1–3]. Abdominal obesity, in particular, substantially increases the risk of developing type 2 diabetes, MetS, and fatty liver. According to the American Association for the Study of Liver Diseases (AASLD), fatty liver in the absence of a chronic increase in alcohol intake (i.e., alcohol intake is <20 g ethanol/day) is referred to as nonalcoholic fatty liver disease (NAFLD) [4]. According to the AASLD's practice guidelines for NAFLD [5], NAFLD is histologically subdivided into nonalcoholic fatty liver (NAFL) and a more severe condition, nonalcoholic steatohepatitis (NASH), which sometimes advances over several decades to life-threatening hepatic cirrhosis and hepatocellular carcinoma. The prevalence of NAFLD, as detected by ultrasound, is up to 30–46% in developed countries and nearly 10% in developing nations, making NAFLD the most common liver disorder worldwide [5, 6].

Lifestyle interventions such as diet and moderate exercise, which lead to weight loss, are fundamental for the treatment of NAFLD. Paradoxically, NAFLD has also been reported in nonobese people [7–9]. In India, individuals with a normal BMI (18.5–24.9 kg/m^2^) have a 2-fold higher risk of developing NAFLD compared with those with a BMI of <18.5 kg/m^2^ [10]. Therefore, NAFLD is expected to become a major burden in Asian countries where the prevalence of obesity is less than that in Western countries [10, 11]. Notably, NAFLD appears to be an early predictor of metabolic disorders, particularly among normal-weight individuals [7]. This is because NAFLD may be more tightly associated with insulin resistance and with markers of oxidative stress and endothelial dysfunction than with the Adult Treatment Panel III criteria for MetS in nonobese, nondiabetic subjects [8]. Therefore, although obese people are predisposed to develop NAFLD, normal weight and overweight people may, through the development of insulin resistance, also show the pathogenic characteristics of NAFLD.

**Figure 1:**
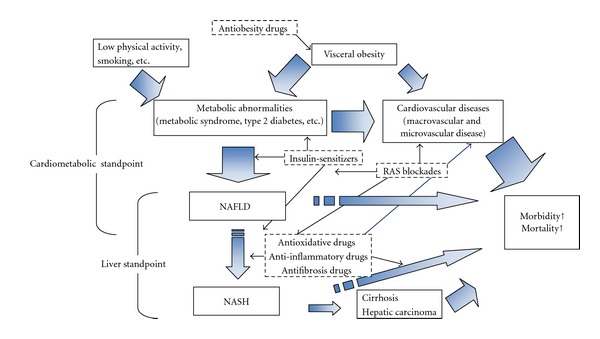
Therapeutic options and their main effects on NAFLD and NASH.

The clinical relevance of NAFLD is still poorly understood because some investigators [12–15], but not all [16, 17], have shown that NAFLD is associated with higher overall mortality and cardiovascular disease. Since NAFLD is closely associated with obesity, diabetes, and MetS, it is unknown whether the relationship between NAFLD and all-cause mortality and cardiovascular death, if any, is independent of cardiometabolic risk factors (Figure 1) such as MetS and type 2 diabetes.

Taken together, NAFLD and NASH are multidisciplinary liver diseases that require interventions targeting the cardiometabolic and liver disorders for the effective treatment of patients with these diseases. Therefore, it is likely that mild NAFLD will require predominantly cardiometabolic pharmacotherapies, whereas moderate to severe NAFLD and NASH will require pharmacotherapies targeting the hepatic disorders. However, since many of the candidate drugs are likely to have broad therapeutic effects targeting multiple aspects of these diseases, distinct classifications are unavailable.

## 2. Liver-Specific Pathogenic Characteristics of NAFLD and NASH

Ectopic fat deposition in organs other than fat tissue, such as the liver and skeletal muscle, reflects severe energy overaccumulation or disturbed fat distribution. However, hepatocytes can, under physiological conditions, store small amounts of triglyceride in a transient manner [18].

Low physical activity as a result of a sedentary state, other unfavorable lifestyle behaviors (e.g., diet and habitual smoking), and sympathetic overdrive as a result of physical/mental stress may lead to insulin resistance independently of obesity. In turn, insulin resistance suppresses the influx of glucose and free fatty acids (FFAs) into adipose tissue, increasing FFA influx into the liver.

The pathogenic characteristics described above are often observed in metabolically obese young women with a normal body weight [19, 20]. Stefan et al. [21] proposed that reduced ectopic fat in the liver may be more important than reduced visceral fat for the discrimination of benign obesity, in other words metabolically normal obesity with good insulin sensitivity.

Ectopic fat deposition in the liver may be an initial feature of peripheral insulin resistance, particularly in adipose tissue, which predominantly accumulates surplus energy as fat. For many years, it was thought that simple steatosis (i.e., fatty liver) remains benign throughout life. However, studies published in the last two decades have shown that such conditions, without appropriate treatment or intervention, provoke other events, including oxidative stress, inflammation, and fibrosis. These events lead to the degeneration and impaired functioning of liver tissue and ultimately result in NASH. Unfortunately, similar to other cardiometabolic diseases, most people with asymptomatic NAFLD remain untreated until the results of blood test or imaging studies indicate the presence of NAFLD. Serum hepatic enzymes, particularly alanine aminotransferase (ALT), are often elevated beyond the normal range [22–24]. However, they may remain within the normal ranges [23, 25] and may be overlooked until abdominal ultrasound, computed tomography, or magnetic resonance imaging are performed. In clinical practice, people with hypertrophic abdominal fat cells often develop NAFLD at a later date. This phenomenon may be caused by direct influx, via the portal vein, of long-chain FFAs, glycerol, and proinflammatory cytokines (e.g., tumor necrosis factor [TNF], interleukin [IL]-6, and IL-1) from the visceral and upper body fat depots, which exhibit insulin resistance and activation of hormone-sensitive lipase and adipose tissue triacylglycerol lipase [26–28].

FFAs derived from the lipolysis of visceral and subcutaneous fat account for approximately 60% of the total hepatic FFA content [29]. Besides increased FFA influx, hyperinsulinemia increases hepatic lipogenesis by activating sterol regulatory element binding protein 1c (SREBP-1c), a key regulator of lipogenic gene expression. Similarly, hyperglycemia caused by inadequate insulin action results in the activation of carbohydrate response element binding protein, which activates L-type pyruvate kinase and lipogenic genes in hepatocytes [30].

Nevertheless, it seems unexpected that hepatic lipogenesis is not attenuated by decreased insulin sensitivity or insulin resistance. Recent studies have suggested that SREBP-1c and lipogenesis are activated secondary to enhanced endoplasmic reticulum (ER) stress. ER stress activates the cleavage of SREBP-1c independently of insulin, which may explain the paradoxical stimulation of lipogenesis in insulin-resistant liver [31]. Consistently, reducing ER stress in obese animals decreases SREBP-1c activation and lipogenesis, and improves hepatic steatosis and insulin sensitivity [32]. Moreover, ER stress in response to increased hepatic lipid content attenuates triglyceride secretion by limiting apolipoprotein B secretion [33], thus increasing hepatic triglyceride accumulation. Alternatively, reduced mitochondrial *β*-oxidation, following inhibition of carnitine palmitoyl transferase-1 (CPT-1), may contribute to enhanced fatty acid synthesis [34].

Several animal studies have also demonstrated a relationship between decreased microsomal triglyceride transfer protein (MTP) expression and steatosis [35]. For example, patients with NASH were reported to show reduced very low-density lipoprotein (VLDL) synthesis and impaired hepatic MTP mRNA expression, which was likely due to single nucleotide polymorphisms of the MTP gene [36].

## 3. Pharmacotherapeutic Candidates for Multidisciplinary Treatment of NAFLD and NASH

In terms of preventing liver damage, the priority for pharmacotherapy of NAFLD is to prevent transformation of NAFLD into NASH and to improve the pathophysiology [5]. Therefore, in theory, antioxidative, antiinflammatory, and antifibrotic agents may constitute first-line treatments for NAFLD. At the same time, treatments should also be initiated to target the cardiometabolic abnormalities and obesity. The potential pharmacotherapies for NAFLD and NASH are listed in Table 1. The order of the drugs is mostly based on the recommendation for use [4–6]. 

### 3.1. Metformin

Metformin (biguanide) is an insulin-sensitizer that is considered to be the first-line treatment of type 2 diabetes because it has relatively few side effects and is inexpensive. Besides reducing hepatic glucose output, metformin also activates AMP kinase, which inhibits the production of glucose, cholesterol, and triglycerides, and stimulates fatty acid oxidation [37].

Nevertheless, the outcomes of clinical trials in NAFLD are conflicting [38, 39]. Metformin was more effective than dietary treatment alone in normalizing several metabolic parameters [40]. Metformin was also better than a prescriptive diet or vitamin E intake for the treatment of NAFLD in patients receiving nutritional counseling [41]. In a clinical study, serum ALT levels were consistently lower in patients treated with metformin compared with those treated with placebo [42]. The effects of metformin in that study were considered to be mediated by weight loss [42].

However, another study showed that metformin only transiently improved hepatic parameters [43]. Moreover, 6 months of treatment with metformin was no better than placebo in terms of improving liver histology in patients with NAFLD [44]. Further well-designed studies are needed to elucidate the significance of metformin treatment for NAFLD.

### 3.2. Thiazolidinediones

A number of clinical trials, including randomized controlled trials, have shown that thiazolidinediones (peroxisome proliferator activated receptor [PPAR]-*γ* agonists) improve liver enzyme levels and liver histology in patients with NAFLD and NASH [39, 45]. PPAR*γ*, which belongs to the nuclear hormone receptor family, is predominantly expressed in adipose tissues and plays a key role in adipogenesis and glucose homeostasis [46]. Since PPAR*γ* is also expressed in cardiovascular tissues, such as vascular endothelial cells, smooth muscle cells, macrophages, and cardiomyocytes, altered PPAR*γ* activity may be involved in the etiology of cardiovascular diseases, particularly atherosclerosis [47].

It has been shown that treatment with pioglitazone for 1-2 years improves the overall pathogenic characteristics of NAFLD and NASH [48–50], suggesting that PPAR*γ* agonists may improve the pathophysiology of the liver as well as clinical factors in patients with NAFLD and NASH. PPAR*γ* regulates several key activities, including adipocyte differentiation and fibroblast differentiation into mature adipocyte types [51, 52]. Therefore, the improvements in NAFLD may be due to improvements in the visceral tissue and upper subcutaneous fat, as thiazolidinediones redirect fat accumulation from the liver or muscle into adipose tissues and confine it in adipose tissue at the expense of adipose cell expansion [53], which unfortunately leads to weight gain.

Thiazolidinediones also activate AMP-activated protein kinase and inhibit lipolysis, at least in part by inhibiting the translocation of hormone-sensitive lipase to lipid droplets [54]. Mayerson et al. [55] showed that treatment with rosiglitazone significantly reduced hepatic triglyceride content and suppresses adipocyte lipolysis. In this way, rosiglitazone increased intramyocellular fat storage as triglycerides, which was accompanied by improved muscle insulin sensitivity. This suggests that intramyocellular fat alone does not necessarily reflect insulin resistance.

However, this apparent improvement in insulin resistance often results in adverse outcomes, such as an increase in the number of adipose cells through enhanced differentiation, eventually leading to weight gain and systematic edema [39, 50]. These events increase the heart's workload and aggravate latent heart failure. Consequently, long-term treatment with thiazolidinediones may be poorly tolerated in some people with diabetes because of their adverse effects, although pioglitazone has fewer side effects than rosiglitazone [56, 57] (troglitazone was excluded from the market because of hepatic toxicity). Some adverse effects of thiazolidinediones, particularly weight gain and edema, could be prevented when administered in low dosages. Indeed, even low-dose pioglitazone (15 mg/day) improved liver enzymes without overt adverse effects in a study of 12 patients with biopsy-confirmed NASH [58].

### 3.3. Vitamin E as an Antioxidative Agent

Some intervention studies in patients with NAFLD have shown that vitamin E, a lipophilic antioxidant, may improve some of the pathogenic characteristics of NAFLD. Vitamin E exerts its antioxidative effects by reducing lipid peroxidation, preventing free radical reactions, and stabilizing cellular phospholipid membranes [59]. Vitamin E also inhibits hepatic transforming growth factor-*β*1 expression, attenuates cytokine stimulation of stellate cells, and protects against hepatic fibrosis [60], suggesting that vitamin E is more beneficial for NASH rather than NAFLD.

Nevertheless, clinical trials performed to date have yielded conflicting results. Some studies, most of them involving small numbers of patients, showed positive outcomes in terms of the treatment of NAFLD [41, 48, 61, 62]. However, other studies showed negative results or no significant improvement with vitamin E compared with placebo or lifestyle interventions, suggesting that vitamin E alone is insufficient to treat NAFLD [38, 63]. Lavine et al. [38] reported that vitamin E and metformin were not superior to placebo in terms of sustained reductions in ALT levels in children with NAFLD. It was also reported that simple lifestyle interventions (i.e., diet and physical exercise) in children with NAFLD can significantly improve liver function, glucose metabolism, and lipid levels beyond those achieved with antioxidant therapy (600 IU/day vitamin E plus 500 mg/day vitamin C) [63]. Several studies have shown that high-dose vitamin E supplementation may be harmful in some patients because of unexpected adverse outcomes, such as increased cardiovascular disease mortality [64, 65], although other studies found no such risk [66, 67].

Among nondiabetic patients with biopsy-confirmed NASH, current recommendations advocate the administration of 800 IU/day vitamin E [5], as this dose may reduce serum ALT levels and improve steatosis, inflammation, and hepatocyte ballooning.

### 3.4. Ursodeoxycholic Acid (UDCA)

UDCA is a cytoprotective antiinflammatory agent that is widely used to treat liver diseases, as well as gallstones. Long-term clinical studies have revealed that UDCA safely and effectively improves hepatic enzyme levels, serum fibrosis markers, and selected metabolic parameters [68]. Oral administration of taurine-conjugated UDCA decreased hepatic steatosis in *ob/ob* mice by cooperatively regulating multiple metabolic pathways, including reduced expression of genes that regulate *de novo* lipogenesis [69]. Overall, however, there were no significant differences between UDCA and placebo in several clinical trials [70].

### 3.5. Long-Chain n-3 Polyunsaturated Fatty Acids (PUFAs)

n-3 PUFAs are mainly consumed in the form of marine oils from fatty fish or other seafood. Fish oil contains both docosahexaenoic acid (DHA, C22:6 n-3) and eicosapentaenoic acid (EPA, C20:5 n-3). n-3 PUFA consumption lowers plasma triglycerides and blood pressure and may reduce inflammation and improve vascular function [71]. These effects occur because n-3 PUFAs are natural ligands for several nuclear receptors and transcription factors that regulate gene expression in various tissues [72]. Considering these properties of n-3 PUFAs, several clinical trials have investigated the potential effects of fish oil or n-3 PUFA consumption on the outcomes of cardiovascular disease. Meta-analyses of these studies have shown that fish oil and n-3 PUFA consumption, but not *α*-linolenic acid, reduce the risk of cardiovascular events and the risk of coronary heart disease-related mortality [71, 73].

Regarding NAFLD, dietary supplementation with long-chain n-3 PUFAs appears to safely reduce nutritional hepatic steatosis in adults [74]. Araya et al. [75] reported that the levels of n-3 PUFAs were decreased while the n-6/n-3 fatty acid ratio was increased in NAFLD patients compared with controls, probably because of defective desaturation of PUFA by inadequate intake of their precursors and increased peroxidation of PUFA. Petit et al. [76] reported that increased erythrocyte n-3 and n-6 PUFA levels are significantly associated with a lower prevalence of steatosis in patients with type 2 diabetes. In addition, the dietary records of middle-aged healthy Japanese men revealed that dietary EPA and EPA + DHA may help to prevent NAFLD [77]. Furthermore, Sato et al. [78] reported that the antiobesity effects of EPA in high-fat/high-sucrose-induced obese mice were associated with the suppression of hepatic lipogenesis and steatosis.

Enjoji and Nakamuta [79] proposed that excess cholesterol intake appears to be one of the main factors associated with NAFLD, particularly in nonobese subjects, because excess cholesterol consumption stimulates the liver X receptor-*α*–SREBP-1c pathway and enhances fatty acid synthesis. Indeed, it was reported that low-dose (2.0% of total energy) fish oil diets improve hepatic lipid accumulation in mice fed a high-cholesterol diet [80]. However, studies examining the effects of EPA/fish oil on dietary cholesterol-induced NAFLD in humans are still lacking.

### 3.6. Statins

Statins (3-hydroxy-3-methyglutaryl-coenzyme A reductase inhibitors) are used worldwide to treat lipid disorders, particularly elevated low-density lipoprotein-cholesterol (LDL-C) and substantially reduce cardiovascular events and mortality. Statins also have pleiotropic effects, including antiinflammatory actions [81], as they greatly reduce the levels of proinflammatory cytokines, such as C-reactive protein (CRP) and TNF. 

Although statins are putatively associated with some adverse events, including elevated hepatic enzymes and liver dysfunction, an elevated serum ALT level at baseline attributable to NAFLD is unlikely to increase the risk of statin-associated elevations in ALT [82]. Similarly, an elevated baseline serum ALT was not associated with an increased risk of hepatotoxicity in patients treated with lovastatin [83].

In clinical studies, simvastatin and atorvastatin [84–86] were associated with a reduction in hepatic steatosis and may inhibit the progression to NASH. Four years of treatment with atorvastatin (20 mg) combined with vitamins C and E reduced hepatic steatosis by 71% in people with NAFLD at baseline [87]. Taken together with a previous report showing that excess cholesterol consumption may accelerate NAFLD [79], statins may be promising candidates for the treatment of NAFLD. Additionally, some statins improved surrogate markers of hepatic steatosis, such as serum glyceraldehyde-derived advanced glycation end-products [88].

On the other hand, considering that statins are associated with worsening of glucose metabolism [89, 90] and that multifactorial medications, such as antihypertensive and antidiabetic drugs, were used in most of the previous studies of statin therapy [91], well-designed, randomized, placebo-controlled studies are needed to determination the suitability of statins as monotherapy or combination therapy for NAFLD.

### 3.7. Fibrates

PPAR*α* is highly expressed in the liver and is involved in fatty acid oxidation [92]. Fibrates, PPAR*α* agonists, increase FFA oxidation in the liver, alter TG synthesis, and reduce hepatic synthesis of VLDL [93, 94], and theoretically improve the pathogenic characteristics of MetS and NAFLD. Unlike the striking outcomes of numerous clinical trials using statins, the results of several large studies of fibrates were inconsistent and had varying outcomes, including the incidence of cardiovascular mortality and events [95–99]. In all of these studies, treatment with a fibrate was associated with a large, although nonsignificant, reduction in cardiovascular events in people with type 2 diabetes or components of the metabolic syndrome.

To date, however, few clinical studies, except for a small pilot study [100, 101], have examined the effects of fibrates on the pathophysiology of NAFLD. Fibrates are expected to ameliorate the pathogenic characteristics of NAFLD because they reduce the levels of inflammatory biomarkers, such as CRP and IL-6, and may improve insulin resistance [102–104], via mechanisms that differ from those of statins. Fenofibrate is commonly used in clinical practice because it is generally well tolerated when used as monotherapy and as combination therapy in a wide range of individuals [103, 105].

No clinical studies have examined the effects or safety of a statin in combination with a fibrate compared with monotherapy for treating NAFLD. However, some benefits of combination therapy on cardiovascular and microvascular outcomes were observed in specific subgroups of patients, such as patients with low HDL levels or hypertriglyceridemia [106].

### 3.8. Niemann-Pick C1-Like 1 (NPC1L1) Inhibitors

NPC1L1 plays a key role in intestinal cholesterol absorption. Ezetimibe, an NPC1L1 inhibitor, was reported to reduce circulating LDL-C levels and improve clinical outcomes in patients at increased risk for cardiovascular events when administered alone or in combination with a statin [107]. Unlike in rodents in which NPC1L1 is mainly expressed in the intestine, NPC1L1 is highly expressed in the liver in humans [108, 109]. This suggests that hepatic NPC1L1 may facilitate hepatic cholesterol accumulation and that ezetimibe may be a potential candidate for NAFLD, especially NAFLD induced by a high-cholesterol diet [79]. In clinical trials, albeit on a small scale, ezetimibe improved biochemical parameters and hepatic enzyme levels, as well as the histological abnormalities of NAFLD [110–112].

### 3.9. Renin-Angiotensin System (RAS) Blockade

The RAS plays key roles in the regulation of blood pressure and fluid balance, as well as in the pathogenesis of insulin resistance and NAFLD [113, 114]. In addition, inhibition of the RAS may improve the intracellular insulin signaling pathway, offering better control of adipose tissue proliferation and adipokine production [115]. The two main classes of RAS blockers, angiotensin II receptor blockers (ARBs) and angiotensin-converting enzyme inhibitors (ACEIs), are efficient drugs that significantly reduce cardiovascular events and mortality [116–118]. As expected, ARBs and ACEIs improve insulin resistance and possibly lipid profiles, suggesting that these agents may be suitable treatments for NAFLD and NASH. Of interest, telmisartan was reported to be a partial agonist of PPAR*γ* [119, 120], a property that does not appear to be shared by other ARBs [121]. Nevertheless, despite many animal studies showing the beneficial effects of ARBs and ACEIs, clinical studies for NAFLD are still lacking.

Beneficial effects of spironolactone and eplerenone, aldosterone antagonists, were recently shown in a mouse model of NAFLD [121–123]. Additionally, spironolactone in combination with vitamin E was reported to improve insulin resistance in patients with NAFLD [124]. However, these effects of aldosterone antagonists have only been shown in mouse and small-scale clinical studies.

Aliskiren, a direct renin inhibitor used to treat hypertension, provides an organ-protective effect by attenuating oxidative stress and improving insulin resistance in mice models [125–127]. Therefore, although aliskiren is a promising drug in terms of improving insulin resistance and oxidative stress, again, human studies are lacking.

### 3.10. Incretin Modulators

Treatment with incretin modulators, glucagon-like peptide-1 (GLP-1) analogs, and dipeptidyl peptidase-4 inhibitors reduces weight gain, minimizes hypoglycemia, decreases inflammation, and is cardioprotective in preclinical studies [128, 129]. The most common adverse events are mild gastrointestinal tract symptoms such as nausea, vomiting, and diarrhea, the incidence and severity of which generally decreased during continued therapy [129].

Regarding the beneficial effects of incretin modulators, weight loss (or weight neutrality) and reduced inflammation appear to be particularly promising for the treatment of NAFLD because these effects facilitate improvements in insulin resistance and metabolic abnormalities. Ding et al. [130] reported that administration of exendin-4, a GLP-1 receptor agonist, induces the regression of hepatic steatosis in *ob/ob* mice by improving insulin sensitivity. GLP-1 appears to protect hepatocytes from fatty acid-related death by suppressing dysfunctional ER stress responses [131]. In addition, GLP-1 suppresses hepatic lipogenesis by activating the AMP kinase pathway and reduces hepatic fat accumulation and nutrient-induced hepatic proinflammatory responses [132]. Tushuizen et al. [133] reported that 44 weeks of exenatide therapy decreased liver fat measured by liver spectroscopy from 15.8% to 4.3% and improved hepatic enzymes in a patient with type 2 diabetes. Taken together, there are promising results in animal models and limited human reports, but clinical studies examining the effects of incretin-based agents on hepatic steatosis have not been performed.

### 3.11. Antiobesity Drugs

Theoretically, improving NAFLD via weight loss is an ideal approach in obese or overweight people because other complications are simultaneously ameliorated. Of several commonly used antiobesity medications [134], orlistat and sibutramine are available for long-term prescription [135, 136]. Orlistat inhibits dietary triglyceride hydrolysis in the gut, occasionally with mild gastrointestinal symptoms, resulting in a substantial decrease in fat absorption. Zelber-Sagi et al. [137] showed that orlistat improves serum ALT levels and steatosis determined by ultrasound in patients with NAFLD, beyond its weight-lowering effects. Harrison et al. [138] reported that subjects who lost ≥5% of their body weight over 9 months experienced improvements in insulin resistance and steatosis, while subjects who lost ≥9% of their body weight also experienced improvements in hepatic histology. 

Sibutramine, a combined norepinephrine and serotonin reuptake inhibitor, reduces food intake and body weight. Both orlistat and sibutramine had beneficial effects on body weight, lipid profiles, glucose metabolism, and inflammatory markers in many trials [135, 137]. However, there is still insufficient safety data regarding the long-term outcomes of antiobesity therapy. Indeed, sibutramine was reported to increase blood pressure and heart rate, which may limit its use in clinical practice [135]. 

Mazindol, a tetracyclic chemical, has been approved in several countries, including Japan. However, long-term treatment with mazindol is not currently permitted, although treatment with mazindol for a few weeks to several months is allowed in severely obese individuals (e.g., BMI ≥ 35 kg/m^2^ in Japan). The combination of mazindol and diet therapy is effective in treating severe obesity [139]. Mazindol exerts antiobesity effects by inhibiting the appetite and activating thermogenesis, as well as having antidiabetic effects [140]. However, clinical studies of mazindol have not been conducted in patients with NAFLD, partly because of the restriction for short-term administration.

Rimonabant, a selective cannabinoid-1 (CB1) receptor blocker, was shown to reduce body weight and improve cardiovascular risk factors in obese patients by regulating the energy balance and body composition [141]. Furthermore, administration of 20 mg rimonabant daily in combination with a hypocaloric diet for 1 year, significantly decreased body weight and waist circumference [142]. Based on several trials showing its weight loss benefits, rimonabant entered the European market for the treatment of obesity [143]. However, the drug was withdrawn worldwide in 2008-2009 because of the emergence of significant side effects, particularly psychiatric disorders (e.g., depression and anxiety) [143, 144]. The CB1 receptor is expressed not only in the central nervous system but also in the gastrointestinal tract, adipose tissue, and cardiovascular system [145]. Consistently, animal studies have shown that CB1 receptor antagonists improve glucose homeostasis, insulin levels, fatty liver, and plasma lipid profiles by blocking the CB1 receptor in peripheral tissues, including the liver and visceral fat [146, 147]. Despite these promising effects, the clinical application of CB1 receptor antagonists will be restricted until their critical adverse effects on the central nervous system can be overcome.

### 3.12. Resveratrol

Polyphenols, ubiquitous dietary components that mainly include flavonoids and tannins, are considered dietary supplements rather than drugs. Several polyphenols obtained from plants may be promising candidate treatments for NAFLD and NASH because they are effective scavengers of reactive oxygen and reactive nitrogen species [148].

Of several polyphenols described to date, resveratrol, a component of several grape species, appears to be particularly relevant in the context of liver disease [149]. For example, resveratrol has antiinflammatory effects mediated through a decrease in proinflammatory cytokines, including TNF. In a study using an animal model of steatosis, resveratrol significantly reduced hepatic steatosis and ALT levels [150]. Resveratrol appeared to reduce liver oxidative stress by increasing the expression of CPT-1a and acyl-coenzyme A oxidase. Another study revealed that resveratrol can also protect the liver from NAFLD by reducing FFA availability [151]. Resveratrol also decreased the severity of NAFLD in rats, at least in part, through its antioxidant effects and by inhibiting TNF [152]. Resveratrol was also reported to protect the liver from NAFLD by enhancing AMP-activated protein kinase phosphorylation [153]. 

### 3.13. Alcohol Intake

Habitual alcohol intake, even light consumption, interferes with the development and progression of critical diseases. Intriguingly, light to moderate alcohol consumption is often associated with a low prevalence of fatty liver. Clinical studies have suggested that light or moderate (<10–20 g ethanol/day) alcohol intake protects against NAFLD [154]. Light alcohol consumption (20 g ethanol on 1–3 days/week or 40–140 g ethanol/week) and moderate alcohol consumption (140–280 g ethanol/week) were independently associated with a low prevalence of fatty liver [155, 156], whereas moderate to heavy drinking (>60 g ethanol/week) was associated with the progression of hepatic steatosis and fibrosis [157, 158].

The effects of moderate alcohol consumption on liver enzymes may increase with increasing BMI [159]. In addition, habitual alcohol consumption generally impairs fatty acid oxidation and stimulates lipogenesis [160, 161]. However, the specific mechanisms by which alcohol (alone or in combination with obesity/metabolic abnormalities) causes liver injury are poorly understood. In clinical studies, the causality between light to moderate alcohol consumption and reduced prevalence of fatty liver or NAFLD remains unknown. Thus, patients with NAFLD should not consume heavy amounts of alcohol [5, 162]. Additionally, no recommendation can be made regarding even light to moderate alcohol consumption [5]. 

## 4. Conclusion

Since hepatic dysfunction is usually closely associated with systemic disorders, there are no specific medications that ameliorate only the pathogenic characteristics of fatty liver without affecting other organs and tissues. In other words, drugs used, or expected to be used, in the treatment of type 2 diabetes, MetS (i.e., hypertension and dyslipidemia), hypercholesterolemia, and obesity may be candidate drugs for the treatment of NAFLD and NASH. These agents can be more effective than monotherapy, although further evidence from animal and cellular studies, as well as large clinical trials, is needed to examine this possibility.

## Figures and Tables

**Table 1:** List of drugs and agents as candidates for the treatment of NAFLD.

Insulin sensitizers	
Metformin (Biguanide)	
Thiazolidines (Pioglitazone, Rosiglitazone)	
Antioxidants	
Vitamin E	
Vitamin C	
Polyphenols (Resveratrol, etc.)	
Hepatocyte-protective agents	
Ursodeoxycholic acid	
n-3 polyunsaturated fatty acids (EPA and DHA)	
Antidyslipidemia	
Statins	
Fibrates	
NPC1L1 inhibitors (ezetimibe)	
RAS blockers	
Angiotensin II receptor blockers	
Angiotensin-converting enzyme inhibitors	
Antialdosterone (spironolactone and eplerenone)	
Renin inhibitor (aliskiren)	
Incretin-related agents	
GLP-1 agonists/analogs (exenatide and liraglutide)	
DPP-4 inhibitors (sitagliptin and vildagliptin)	
Antiobesity drugs	
Orlistat	
Sibutramine	
Mazindol	
